# Molecular Characterization and Clinical Implications of Spindle Cells in Nasopharyngeal Carcinoma: A Novel Molecule-Morphology Model of Tumor Progression Proposed

**DOI:** 10.1371/journal.pone.0083135

**Published:** 2013-12-12

**Authors:** Weiren Luo, Kaitai Yao

**Affiliations:** Cancer Research Institute, Southern Medical University, Guangzhou, PR China; Institute of Cancerology Gustave Roussy, France

## Abstract

Up to now, the precise molecular and morphological changes underlying the invasive and metastatic properties of nasopharyngeal carcinoma (NPC) remain largely unresolved. We speculate that neoplastic spindle cells, which are prominently found in the invasive tumor front and the surrounding stroma, might be responsible for the aggressive patterns. Expression profiling of various biomarkers relevant to cancer stem cells (CSCs) and epithelial-mesenchymal transition (EMT) was performed by tissue microarray-based immunohistochemistry in NPC samples. The expression of EBER and LMP1 was detected by in situ hybridization and immunohistochemistry, respectively. We found that overexpression of CSCs-related markers (ALDH1, Nanog and ABCG2) and up-regulation of EMT markers (Fibronectin, MMP-2, Periostin, SPARC, Snail and Slug), together with E- to N-cadherin switching, occurred preferentially in tumors containing a large proportion of spindle-shaped malignant cells. Furthermore, CSCs-like properties were highly present in spindle cells compared with non-spindle cells of tumors, and correlated strongly with EMT features. In addition, EBV-related factors EBER and LMP1 were highly expressed and correlated strongly with CSCs and EMT characteristics in neoplastic spindle cells. Importantly, high proportion of spindle cells (≥20%) correlated significantly with various aggressive aspects including lymph node metastasis (*P* = 0.031) and local recurrence (*P* = 0.014). Patients with high proportion of spindle cells had poor survival (*P* = 0.004), though it was not an independent value. In conclusion, we demonstrate that spindle cells could be valuable morphological indicators of tumor progression and unfavorable prognosis of NPC. An integrated molecule-morphology model of NPC firstly constructed may shed significant light on the metastatic cascade and clinical relevance of patients.

## Introduction

Epithelial-mesenchymal transition (EMT), known as the loss of epithelial differentiation and acquisition of the mesenchymal phenotype, is crucial for morphogenesis during embryonic development [1]. In the past few years, EMT is thought to be critical to tumor progression and metastasis [2,3]. During the processes of EMT, malignant cells of epithelial origin lose their epithelial polarity, and generate the more migratory and invasive capabilities. On the other hand, a small subpopulation of cancer cells capable of self-renewing and tumorigenesis, that is called cancer stem cells (CSCs), are responsible for tumor initiation and recurrence [4,5]. Interestingly, growing evidence demonstrates that cells which acquire stem cell-like properties undergo EMT and then establish highly mobile and invasive abilities [6-8]. For this reason, they have also been termed as ‘‘mobile/migratory cancer stem cells”, which was initially proposed by Brabletz T et al in colorectal cancer patients through the method of immunohistochemistry [9].

Nasopharyngeal carcinoma (NPC) is a disease with remarkably distinctive ethnic and geographic distributions, which is highly prevalent in Southern China and Southeast Asia [10]. Unlike other head and neck cancers, NPC frequently metastasizes to regional lymph nodes when diagnosed and has a predilection for the development of local recurrence after therapy [11,12]. However, the precise molecular and morphological changes responsible for its high-aggressive potential remain largely unknown. Microscopically, the most common subtype of NPC is categorized as non-keratinizing carcinoma, which is divided into two main sbutypes: one of which is descirbed as differentiated carcinoma (DNKC) (the subtype is characterized by cellular stratification and often with a well-defined borders, we here call “non-spindle cells”), and the other is referred to undifferentiated carcinoma (UDC) (this subtype is defined as syncytial-appearing large cancer cells with indistinct cell borders) [13]. It is noteworthy that either the differentiated or the undifferentiated subtype generally comprises a certain content of mesenchymal-like cell (that is “neoplastic spindle cells”), which is predominantly observed in the invasive front (tumor-host interface) and tumor stroma [14-17]. Recently, our findings show hat EMT markers E-cadherin expression was mostly inhibited in the spindle cells, whereas the expression of vimentin, fibronectin, Snail and Slug was upregulated [16]. We suggest that EMT may be crucial for the pathogenesis of neoplastic spindle cells in NPC, and spindle cells should be considered as the more aggressive subtype. However, up to now, the molecular and biological patterns of neoplastic spindle cells still remain poorly understood.

The purpose of the present study was further to investigate whether these malignant spindle cells have invasive and metastasic properties of CSCs in NPC patients. To this end, expression profiling of various CSCs and EMT-related biomarkers was performed in 122 NPC samples. One of remarkable findings of this study is that, spindle cells might be valuable morphological markers for tumor aggressiveness and poor prognosis of NPC. At last, an integrated molecule-morphology model was constructed to elucidate tumor progression and metastasis of this disease. 

## Materials and Methods

### Patients and Tissue Samples

A series of 122 human primary NPC tissues between 2003 and 2005 were obtained from the Department of Pathology, the People's Hospital of Gaozhou City, China [18]. No patients received preoperative therapy. The formalin-fixed paraffin-embedded human NPC tissues were anonymous. Written informed consent for the tissue samples has obtained from all patients. This consent procedure and this study were approved by the Guangdong Medical College Institutional Research Ethics Committee. Pathologic staging was done based on the TNM classification (AJCC/UICC 2002). The complete follow-up was finished on October 26, 2010, and the median follow-up was 51.9 months (range, 8 to 92 months). The clinicopathologic variables were shown as described previously [18]. Histological subtypes were determined according to the World Health Organization (WHO) classification. All cases were classified as non-keratinizing carcinoma. Microscopically, the 18 DNKC tumors comprised non-spindle cell (with epithelial-like phenotype) and spindle cell components (with mesenchymal-like morphology) and the 97 UDC tumors were composed of malignant syncytial-appearing large cells and spindle cells. Additionally, 29 of the UDC tumors also comprised non-spindled neoplastic epithelial cells. In total, there was a neoplastic spindle cells component in 115 cases (range, 10% to 90%), and a non-spindled cells component in 47 cases (range, 5% to 85%) [16].

### Tissue Microarray (TMA) Construction and Immunohistochemistry

One core with a diameter of 1.5 mm was obtained from the representative area of each case carefully selected on H&E-stained sections and inserted into new paraffin blocks using a TMA workstation (Beecher Instruments, Silver Spring, MD). At last, sections from these blocks were cut into 4 µm thick. Immunohistochemical staining was performed according to the standard streptavidin-peroxidase (SP) method (Zymed, San Francisco, CA). Briefly, sections of sequential TMA sections were dewaxed, and rehydrated. Antigen retrieval was performed by high-pressure in citrate buffer (PH 6.0) and boiled for 2 min. Endogenous peroxidase and non-reactive staining were blocked by 3% H_2_O_2_ and 1% BSA for 15 min at room temperature, respectively. The sections were then incubated overnight at 4°C with primary antibodies. Antibodies and conditions used are summarized in the Table S1. 

To assess IHC results, the proportion of tumor cell staining was graded as follows: 0, no positive tumor cells; 1, <10% positive tumor cells; 2, 10-50% positive tumor cells; 3, >50% positive tumor cells. As well, the staining intensity of tumor cell was graded as follows: 0, no staining; 1, weak staining; 2, modest staining; 3, strong staining. The final score was calculated by multiplying the intensity scores with staining area scores (0, 1, 2, 3, 4, 6, 9). The final staining scores ≤ 4 and ≥6 were regarded as tumors with low and high expression of EMT/CSCs-related markers and LMP1, respectively [18,19]. On the other hand, immunohistochemical biomarkers in the spindle cells and non-spindle carcinoma cells components were considered to be positive reaction when >10% of tumor cells were immunoreactive [16]. In addition, 115 cases with a neoplastic spindle cells component were clustered into two groups based on the percentage of spindle cell at 20%, a value derived from cutoff analysis of clinicopathological parameters and survival in patients with NPC. Those cases with spindle cells < 20% were considered to be NPC with low proportion of spindle cells, and those with spindle cells ≥ 20% were regarded as NPC with high proportion of spindle cells [20]. 

Correlations were examined by χ^2^ test between variables. Overall survival of patients was determined using the Kaplan-Meier method and the log-rank test. Unsupervised hierarchical clustering analysis was performed using Cluster 3.0, and graphical representation of the clustering was done with TreeView. Statistical analyses were performed using the SPSS 13.0 software (SPSS Inc, Chicago, IL). *P* values <0.05 were considered statistically significant.

### Cell Culture, Immunofluorescence and Western Blot

Human NPC cell lines epithelial-like CNE-1 and spindle shaped-like C666-1 cells [21] were grown in RPMI 1640 culture medium containing 100 units/ml of penicillin, and 100 μg/ml streptomycin in a humidified 5% CO2 atmosphere at 37°C. For immunofluorescence analysis, cells were grown on sterile 12-mm glass coverslips for 24 h at 37°C. After washing twice with PBS, they were fixed in 4% paraformaldehyde (Sigma) for 30 min at room temperature. After washing with PBS, cells were incubated 2 h at room temperature with the appropriate primary antibodies including E-cadherin (1:500), N-cadherin (1:100), Vimentin (1:400), ALDH1 (1:200), OCT4 (1:50), SOX2 (1:300) and Nanog (1:600). The following operation steps were the same as described previously [22]. At last, cell nuclei were counterstained with DAPI (Boisynthesis biotechnology, Beijing, China) and representative images were examined using a confocal microscope (FV300 Olympus, Tokyo, Japan). As previously described [22], total cell proteins were extracted and protein concentration was determined by using BCA assay (Beyotime Inc, China) for Western blot analysis. Membranes were immunoblotted overnight at 4°C with the diluted primary antibodies ALDH1 (1:500), OCT4 (1:100), SOX2 (1:600) and Nanog (1:1000). Signals were detected using enhanced chemiluminescence (ECL) reagents (Bio-Rad, USA).

### Quantitative RT-PCR (qRT-PCR)

Total RNA of C666-1 and CNE1 cells was reversely transcribed using PrimeScript® RT reagent Kit (TaKaRa). Quantitative real-time reverse transcription-PCR (qRT-PCR) was performed with SYBR® Premix Ex Taq™ II (TaKaRa) on a StrataGene Mx3005p System, following the manufacturer’s instructions. The primers (5’ to 3’) were：OCT4 forward primer: TATTCAGCCAAACGACCATC and reverse primer: GCCTCTCACTCGGTTCTC; SOX2 forward primer: CGAGTGGAAACTTTTGTCGGA and reverse primer: TGTGCAGCGCTCGCAG; Nanog forward primer: TGTCTTCTGCTGAGATGCCTCACA and reverse primer: CCTTCTGCGTCACACCATTGCTAT; ALDH1 forward primer: ACGCCAGACTTACCTGTCCTAC and reverse primer: TCCTCCTTATCTCCTTCTTCTACCT. Quality of different cDNA samples were normalized by using GAPDH as a housekeeping gene (forward primer, GAGTCCACTGGCGTCTTC; reverse primer, GATGATCTTGAGGCTGTTGTC). These data were repeated on three independent experiments.

### In Situ Hybridization (ISH) for EBV-Encoded RNA

Before detecting the staining of EBER, poly d(T) oligonucleotide probes (strong nuclear staining in all of the samples) were undertaken to make sure that tissue RNA in the formalin-fixed paraffin-embedded sections was not degraded. In situ hybridization of EBER was detected as previously described [23,24]. Detection of EBERs was carried out using a digoxigenin-labeled antisense EBER kit (ZSGQ, Beijing, China). After being deparaffinized, rehydrated, sections were predigested with proteinase K (1:30) and hybridized to the digoxigenin-conjugated probes. Subsequently, sections were incubated with anti-digoxigenin conjugated with alkaline phosphatase for detection of hybridization. The hybridization signals were visualized with 3,3′-diaminobenzidine (DAB) and slides were counterstained with eosin. In each run, a sense probe for EBER was used as the negative control. EBER has been shown to be positive in nearly all non-keratinizing NPCs [23,24]. As previously reported, the assessment was always based on percentage of stained tumor cells as follows: 0 (negative), 1 (<10%), 2 (10-50%), or 3 (>50%) [25]. Totally, the score as 1, 2, or 3 was considered as EBER-positive. In order to show the differentially expressed levels of EBER between neoplastic spindle cells and non-spindled cells, in the present study, the score of EBER expression was determined based on both the intensity and the percentage of positive tumor cells. According to the intensity, staining in the nuclei of cancer cells was determined as the positive hybridization signal. The definition of staining intensity was described below: 0 = no staining, 1= yellow staining, 2 = dark brown. On the other hand, the assessment of percentage of stained tumor cells was described as above. Taken together, tumor cells with dark brown staining that scored 1, 2, or 3 were considered as EBER-high expression in NPC. 

## Results

### Expression Profiling of Biomarkers Belong to CSCs and EMT Identifies Two Main Subgroups of NPC

**Figure 1:**
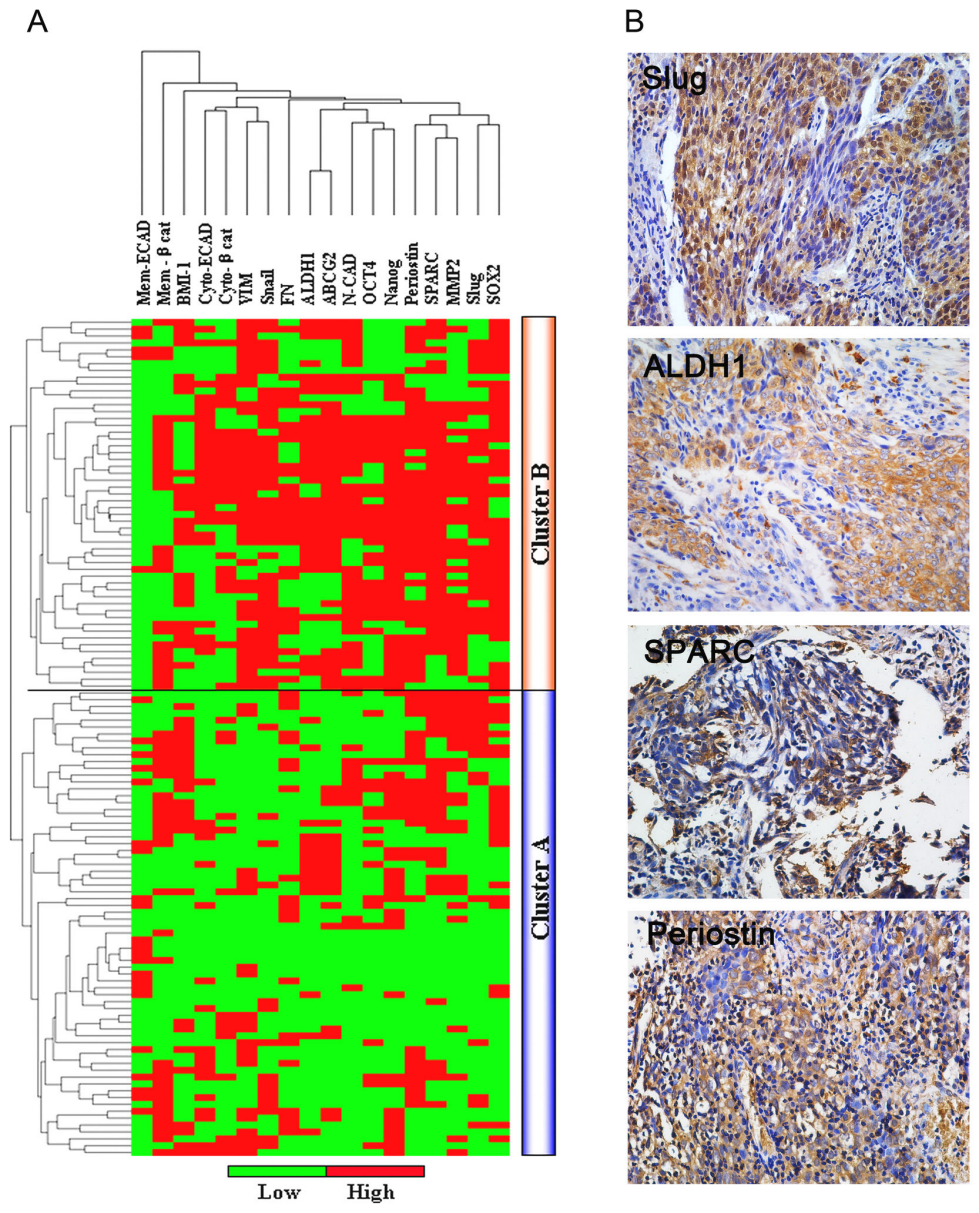
EMT and CSCs-associated markers are predominately expressed in the main subgroup of NPC. (A) Unsupervised clustering of 18 proteins in 122 NPC (non-keratinizing carcinoma) samples classified phenotypically distinct subgroups of tumors. Rows, experimental samples; columns, individual genes. High expression levels are in red and low expression levels in green, as indicated in the scale bar below. Tumors were divided into two major clusters (cluster A and B, segregated by a black line). Elevated expression of EMT and CSCs-related markers occurred more frequently in Cluster B than Cluster A in tumors. Of note, the majority of the tumors in cluster B displayed a spindle-shaped phenotype (≥20%). (B) Immunohistochemical staining of selected markers in tumors, especially in the spindle cells component.

First of all, we evaluated the expression profiling of immunomarkers associated with CSCs and EMT in a series of 122 non-keratinizing NPC tissues. The details of 16 biomarkers are summarized in Table S1. Unsupervised hierarchical clustering subdivided the tumors in two main groups (cluster A and B) (Figure 1A). We found that cluster B showed enrichment of CSCs-related markers (ALDH1, SOX2, OCT4, Nanog and ABCG2). A cadherin switching (decreased expression of E-cadherin and overexpression of N-cadherin), as well as overexpression of other EMT markers (Fibronectin, MMP-2, Periostin, SPARC, Snail and Slug), was also preferentially occurred in cluster B (Table S2). In comparison, cluster A had low expression of these CSCs (ALDH1, SOX2, OCT4, Nanog and ABCG2) and EMT-associated (N-cadherin, Fibronectin, MMP-2, Periostin, SPARC, Snail and Slug) biomarkers. Of interest, tumors with a phenotype of spindle cells (≥20%) were also more frequently present in cluster B compared with those in cluster A (65% *vs* 25.0%) (Table S2). Based on these findings, we indicate that there may be a possible link between neoplastic spindle cells and the occurrence of CSCs and EMT in tumors

### Neoplastic Spindle Cells in NPC Show Invasive Properties of CSCs

Subsequently, we found that tumors containing more than 20% spindle cells showed significantly up-regulation of CSCs-like proteins (ALDH1, Nanog and ABCG2) (Table 1). In addition, reduction of membranous E-cadherin and elevated expression of other EMT markers (N-cadherin, Fibronectin, MMP-2, Periostin, SPARC, Snail and Slug) occurred predominately in spindle cells group (*P* <0.05) (Table 1). 

**Table 1:** The significant expression of CSCs and EMT biomarkers in 122 NPCs with neoplastic spindle cells.

Variables	*n*	Tumors with spindle cells		
		<20% (*n*, %)	≥20% (*n*, %)	*P*
ALDH1				
Low expression	74	51 (69)	23 (31)	0.001
High expression	48	19 (40)	29 (60)	
Nanog				
Low expression	51	35 (69)	16 (31)	0.033
High expression	71	35 (49)	36 (51)	
ABCG2				
Low expression	64	45 (70)	19 (30)	0.002
High expression	58	25 (43)	33 (57)	
E-cadherin (Membrane)				
Low expression	95	50(53)	45 (47)	0.047
High expression	27	20 (74)	7 (26)	
E-cadherin (Cytoplasm)				
Low expression	76	52 (68)	24 (32)	0.002
High expression	46	18 (39)	28 (61)	
N-cadherin				
Low expression	67	44 (66)	23 (34)	0.041
High expression	55	26 (47)	29 (53)	
β-catenin (Cytoplasm)				
Low expression	82	53 (65)	29 (35)	0.020
High expression	40	17 (42)	23 (58)	
Fibronectin				
Low expression	73	49 (67)	24 (33)	0.008
High expression	49	21 (43)	28 (57)	
MMP2				
Low expression	65	47 (72)	18 (28)	0.000
High expression	57	23 (40)	34 (60)	
Periostin				
Low expression	52	49 (94)	3 (6)	0.000
High expression	70	21 (30)	49 (70)	
SPARC				
Low expression	50	40 (80)	10 (20)	0.000
High expression	72	30 (42)	42 (58)	
Snail				
Low expression	62	43 (69)	19 (31)	0.007
High expression	60	27 (45)	33 (55)	
Slug				
Low expression	69	49 (71)	20 (29)	0.001
High expression	53	21 (40)	32 (60)	

CSCs, cancer stem cells; EMT, epithelial-mesenchymal transition; ALDH1, aldehyde dehydrogenase 1.

**Figure 2:**
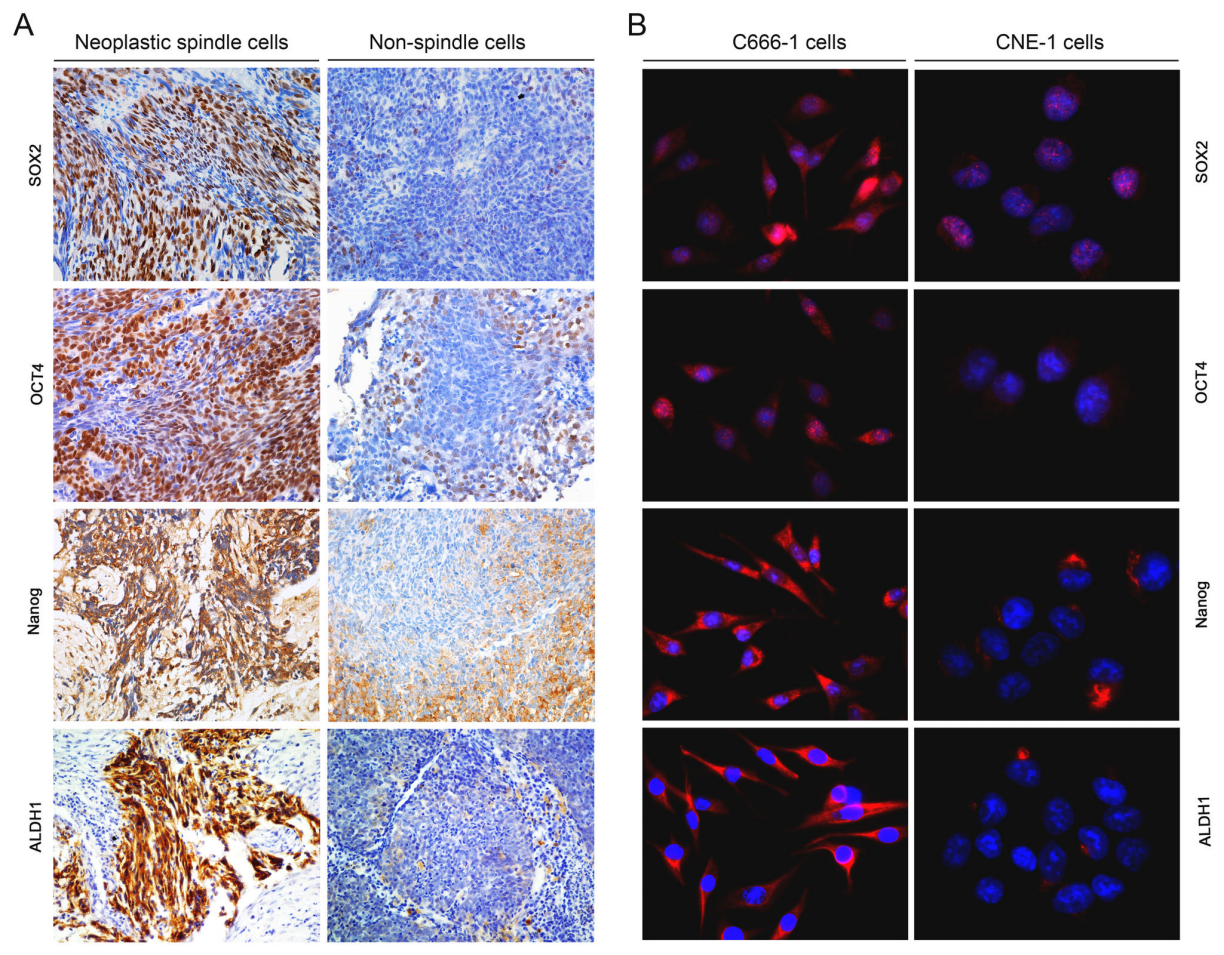
Neoplastic spindle cells exhibit properties of stem cells. (A) Stem cells-like markers SOX2, OCT4, Nanog and ALDH1 were markedly expressed in neoplastic spindle cells compared with neoplastic non-spindle cells (DNKC) in NPC. (B) Different expression levels of SOX2, OCT4, Nanog and ALDH1 proteins in human NPC C666-1 (undifferentiated, spindle-like morphology) and CNE1 (well differentiated, epithelial-like morphology) cell lines detected by immunofluorescence analysis.

**Figure 3:**
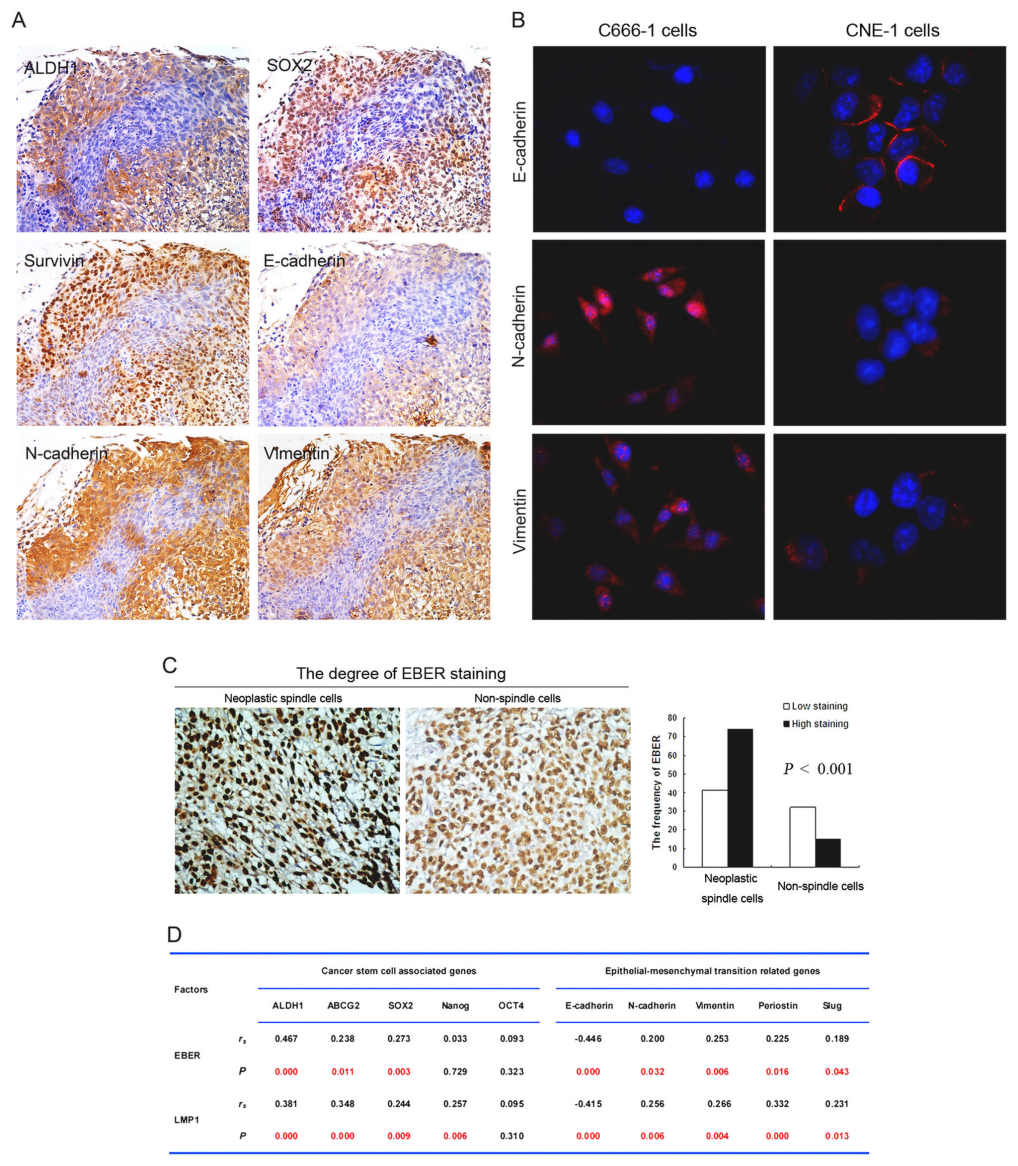
Stem-like spindle cells are strongly associated with EMT -like phenotypic changes and EBV infection. (A) Serial sections from the same patient showed aberrant expression of EMT indicators E-cahderin, N-cadherin and Vimentin in stem-like spindle cells. (B) Spindle-like C666-1 cells had strong fluorescence staining of N-cadherin and Vimentin, and weak staining of E-cadherin compared with epithelial-like CNE-1 cells. (C) Representative images show that high expression (dark brown) of EBV-encoded small RNA (EBER) was observed in neoplastic spindle cells in comparison with non-spindle cells in the same tumor. (D) The significant relationship was found between EBER, EBV-encoded protein LMP1 and CSCs/EMT-related markers. Differences statistically significant at *P* values <0.05.

To further confirm the concept that neoplastic spindle cells possess the invasive phenotype of CSCs, the expression of CSCs and EMT markers between the neoplastic spindle cells (115 cases) and non-spindle cells components (47 cases) of 122 tumors was analyzed. Compared with the non-spindle cells component of the tumors, stem-like markers SOX2, OCT4, Nanog, and ALDH1 [16] were significantly expressed in neoplastic spindle cells component (Table S3 and Figure 2A). In addition, Survivin overexpression was found more frequently in neoplastic spindle cells (96/115, 83%). In contrast, in neoplastic non-spindle cells, 79% of cases (37/47) showed weak or negative staining for this protein (Table S3). On the other hand, overexpression of EMT markers together with the cadherin switching was also more frequently present in the spindle cells. And some markers have previously been described [16]. Of interest, there was a significant correlation between the expression levels of CSCs and EMT in neoplastic spindle cells. For instance, Spearman correlation analysis showed that ALDH1 expression in spindle cells correlated negatively with E-cadherin expression (*rs* = -0.567, *P* < 0.001), whereas positively with N-cadherin (*rs* = 0.288, *P* = 0.002) and Vimentin expression (*rs* = 0.449, *P* <0.001), as shown in Figure 3A.

### The Spindle-Shaped Cell Line C666-1 Exhibits Features of CSCs and EMT

Immunofluorescence results showed that expression of SOX2, OCT4, Nanog and ALDH1 was markedly displayed in mesenchymal-like C666-1 cell line in comparison with epithelial-like CNE1 cell line (Figure 2B). Western blot analyses and qRT-PCR analyses also verified these results (data not shown). O the other hand, C666-1 cells showed a strongly reduced expression of E-cadherin, and a significantly increased expression of N-cadherin and Vimentin, compared with the expression levels of those in CNE1 cells (Figure 3B).

### High Levels of EBER and LMP1 Staining in Neoplastic Spindle and Correlate Significantly with CSCs and EMT Properties

Similar to other observations [23-25], we found that EBER was positive in all non-keratinizing NPCs according to the percentage of stained tumor cells. Of note, we found that there was considerable variation in hybridization intensity between a spindle cells and non-spindle cells component of tumors. To make it sense, we defined positive tumor cells with dark brown hybridization signal as high expression of EBER in NPC tissues. Of 115 cases with neoplastic spindle cells, 63% of samples (74/115) showed high expression of EBER in the spindle cells. In contrast, of 47 samples with non-spindle cells, 68% of cases (32/47) exhibited weak staining of EBER in the non-spindle cells (*P* < 0.001) (Figure 3C). Meanwhile, in 115 cases with neoplastic spindle cells, 57 cases (50%) displayed high expression of EBV-encoded protein LMP1 in the spindle cells. In contrast, of 47 neoplastic non-spindle cells samples, this protein was only found in 9 cases (19%) (Table S3). Furthermore, in the neoplastic spindle cells, Spearman correlation analysis showed that EBER and LMP1 correlated significantly with the expression of CSCs and EMT-related proteins (*P* <0.05) (Figure 3D). 

### Correlation between Neoplastic Spindle Cells and Clinicopathological Parameters and Survival in NPC

**Figure 4:**
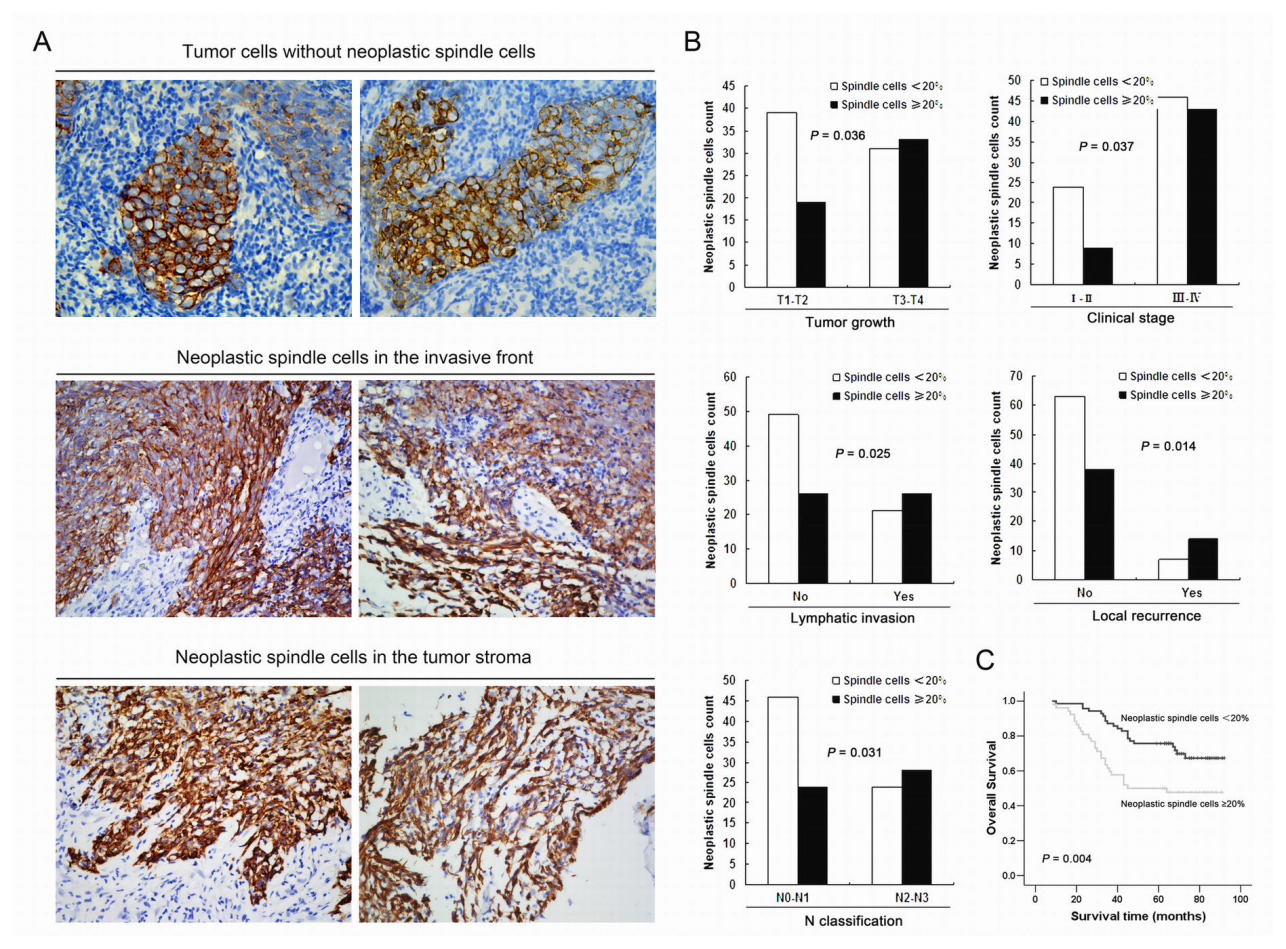
Spindle cells contribute to tumor aggressiveness and poor survival in NPC. (A) Representative examples showed that neoplastic spindle cells were frequently located at the invasive tumor edge or the invading surroundings (Pan-cytokeratin staining). (B) Correlation between the clinicopathologic characteristics and spindle cells in NPC. Columns: number of cases with >20% spindle cells vs <20. (C) Cumulative overall survival curves of 122 NPC patients according to the degree of spindle cells (≥20% vs <20%). Patients with high percentage of spindle cells (≥20%) had significantly shorter overall survival. *P*-values were calculated by the log-rank test.

Spindle cells were frequently observed in the invasive margin of tumors or cancer cells infiltrating the adjacent stroma (Pan-cytokeratin staining; Figure 4A). To test whether spindle cells contribute to aggressive behaviors during NPC progression, the association of clinicopathological features with spindle cells was analyzed. We showed that neoplastic spindle cells (≥20%; cut-off point) was positively associated with aggressive behaviors including T classification (*P* = 0.036), lymphatic invasion (*P* = 0.025), lymph node metastasis (*P* = 0.031), advanced clinical stage (*P* = 0.037) and local recurrence (*P* = 0.014) (Figure 4B). Spearman correlation analysis was used to further confirm these findings.

Regarding overall survival, the log-rank test showed that patients with high percentage of spindle cells (≥20%) showed a significantly reduced overall survival time when compared with those with low percentage of spindle cells (*P* = 0.004) (Figure 4C). Specifically, patients with high percentage of spindle cells (*n* = 52) had a median survival time of 58.9 months (95% confidence interval, 50.2-67.6), whereas it was 76.3 months (95% confidence interval: 70.5-82.2) for patients with low percentage of spindle cells (*n* = 70). Furthermore, the cumulative 5-year survival rate in patients with low percentage of spindle cells was 76% (95% confidence interval, 0.656-0.858), whereas it was 50% (95% confidence interval, 0.364-0.636) in the high proportion group. When including individual variables in a Cox proportional hazards model, the degree of spindle cells had a significantly prognostic effect on overall survival of patients (95% confidence interval, 1.283-4.025; *P* = 0.005). However, in a final simultaneous model, the spindle cells was not independent prognostic factor for NPC patients (95% confidence interval, 0.852-2.981; *P* = 0.144).

## Discussion

Neoplastic spindle cells are frequently located in the tumor invasive front [14-16,26]. However, the molecular and biologic characteristics of these tumor cells are not fully defined. Tissue microarray (TMA) is an effective tool for high-throughput molecular analysis of tumors [27]. With the development of expression profiling and clustering analysis, TMA-based immunostaining data has been well established to identify different subgroups in various tumors [28]. We here took advantage of TMA method and hierarchical clustering in a series of 122 NPC cases using 16 involved immunohistochemical markers. Interestingly, our findings showed that high expression of CSCs markers (ALDH1, Nanog and ABCG2) as well as EMT markers (Fibronectin, MMP-2, Periostin, SPARC, Snail and Slug), together with a shift from E- to N-cadherin expression, occurred preferentially in primary tumors with a large proportion of spindle cells (≥20%). Thus, we suggest that there might be a possible relationship between the EMT/stem-like signature and spindle cells in NPC tissues. Similar to our findings, Sarrió D et al. conducted a TMA-based immunohistochemical study in 479 invasive breast carcinomas and found that EMT-like changes occurred predominantly in the basal-like subtype, the poorly differentiated phenotype of breast cancer [29,30]. In addition, the similar signatures have also been identified in other human tumors [31,32]. 

To further determine whether these spindle-shaped cells possess CSCs features, the expression levels of relevant biomarkers were examined in the neoplastic spindle cells and non-spindle cells components of NPC. Aldehyde dehydrogenase 1 (ALDH1) has been employed successfully to isolate CSCs from several cancer cell lines including breast cancer, pancreatic cancer, lung cancer, prostate cancer, and colon cancer [33-37]. SOX2, OCT4 and Nanog, the key hallmarks of embryonic stem cells (ESCs), have been confirmed to have a strong relationship with the induction of CSCs [22,38-40]. Interestingly, we found that neoplastic spindle cells highly expressed ALDH1 in comparison with non-spindle cells. Similarly, overexpression of ESCs markers occurred more frequently in neoplastic spindle cells in this study. Supporting our results, it has provided evidence that, at the histopathological level, undifferentiated counterparts of breast cancer contained higher proportions of stem cell-like characteristics [41,42]. One of the remarkable properties of CSCs is highly resistant to apoptosis [4,5]. We observed that the expression of Survivin, a member of the family of inhibitor of apoptosis proteins (IAP), was markedly increased in malignant spindle cells. Supporting our findings, Harn HJ et al. reported that few apoptotic cells were observed in the spindle cells type of NPC by TUNEL method [43]. Taken together, we indicate that neoplastic spindle cells might be a distinct subpopulation of cells enriched for stem cell activity in NPC patients.

Epithelial-mesenchymal transition (EMT) is defined as the loss of epithelial plasticity and by switching toward mesenchymal phenotype during tumor progression [2,3]. A cadherin switch (from E-cadherin to N-cadherin) and the mesenchymal marker Vimentin have been reported to correlate strongly with EMT and convert cancer cells into invasive phenotype [44-46]. Our data showed that these EMT signatures occurred more frequently in spindle cells with CSCs features. For example, up-regulation of ALDH1 in neoplastic spindle cells correlated inversely with reduced E-cadherin expression, and positively with overexpression of N-cadherin and Vimentin. In addition, we found that human NPC cell line C666-1, which generally displays a fibroblast-like, spindle-shaped morphology, generated features of CSCs and EMT *in vitro*. Collectively, we demonstrate that these stem-like spindle cells might closely resemble cells with invasive potentials. Accordingly, Weinberg and other groups have recently demonstrated that epithelial stem cells could endow cells with invasive properties through EMT induction [7,47]. Indeed, evidence connecting EMT to the presence of CSCs has initially been explored by Brabletz T et al in colorectal cancer tissues, and these cells were termed as the notion of “mobile/migratory CSCs” [9]. Therefore, we propose that stem-like spindle cells with invasive properties of EMT might likely to be “mobile/migratory CSCs” in NPC. 

On the basis of these data, we speculate that neoplastic spindle cells might contribute to tumor aggression of NPC. As anticipated, neoplastic spindle cells correlated significantly with various aggressive aspects of NPC patients. For example, high proportions of malignant spindle cells (≥20%; cut-off point) were more frequently present in patients with T3-T4 classification than those with T1-T2 classification, suggesting that cancer cells with more spindle cells acquire highly mobile capabilities that enable invasion of the extracellular matrix. We suggest that EMT may contribute directly to the aggressive clinical behavior of spindle cells during NPC progression. Of note, tumor relapse was found to be more frequently developed in patients with high percentage of spindle cells. Growing evidence shows that CSCs are responsible for tumor recurrence [4,5]. Thus, our findings further confirm that the acquisition of stem-like properties may occur in these spindle cells. Overall, we suggest that spindle cells are important morphological indicators, indicating more aggressive clinical behaviors and poor prognosis of NPC.

The molecular mechanisms responsible for triggering spindle cells in NPC remain unclear. It is well known that Epstein-Barr virus (EBV) infection is crucial for the pathogenesis and differentiation of NPC [23]. Several reports have showed the detection of EBER only in patients with non-keratinizing NPC [48]. Of note, our findings further reveal that high expression levels of EBER and LMP1 occurred predominately in neoplastic spindle cells in comparison with non-spindle cells in non-keratinizing NPC. Moreover, the significant relationship between these two EBV-related factors and EMT/CSCs properties was observed in the spindle cells components of tumors. Supporting our findings, several studies have revealed that ectopic expression of LMP1 could promote an invasive mesenchymal-like appearance and generate EMT and stem-like features in NPC cell lines [49-51]. Taken together, EBV oncoprotein LMP1 may be the dominant cause of facilitating the histogenesis and aggressiveness of spindle cells in NPC. 

**Figure 5:**
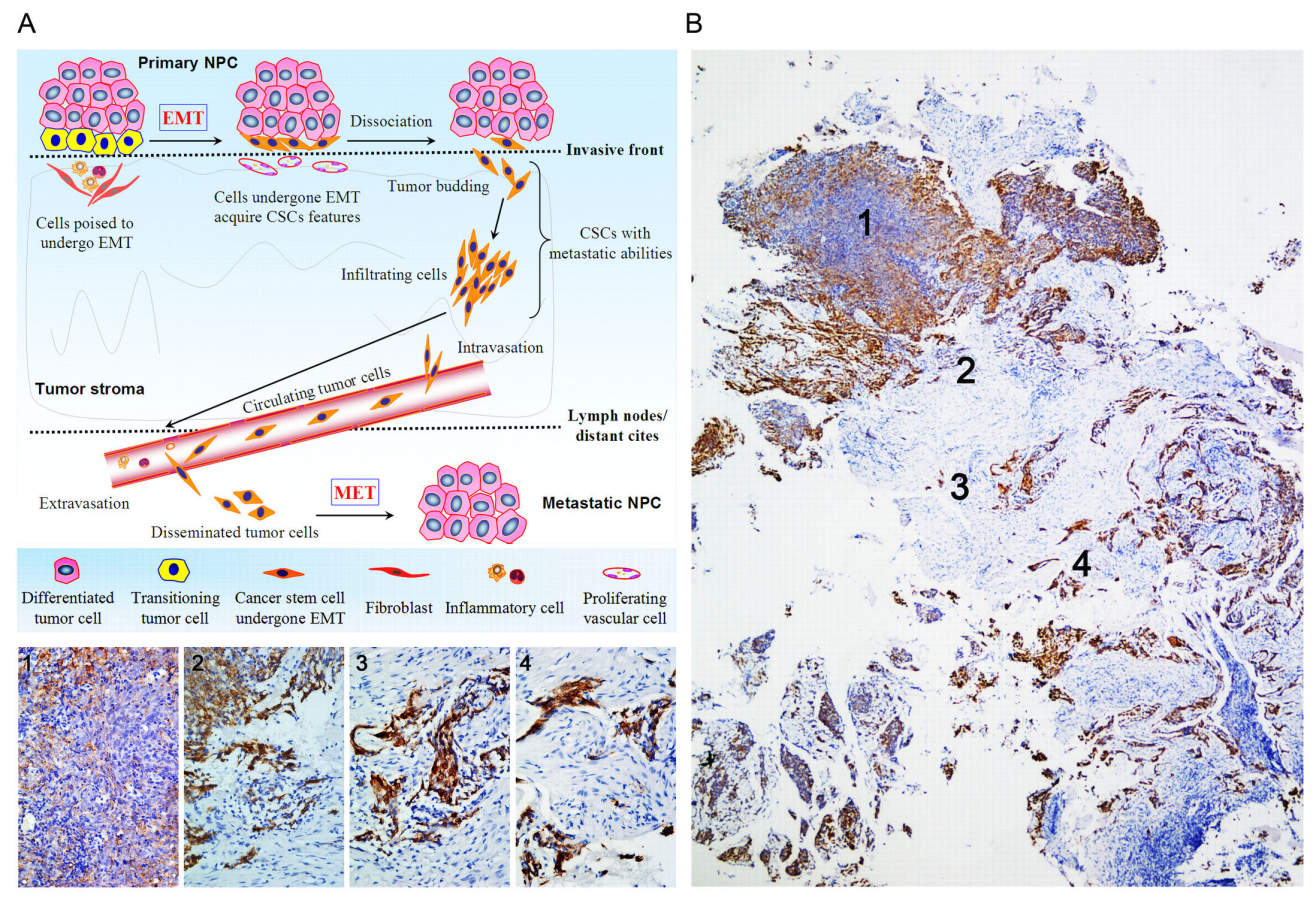
A molecule-morphology model of tumor progression and metastasis in NPC firstly put forward. (A) The diagram depicts that spindle cells with CSCs and EMT characteristics are crucial for NPC aggressive progression. (B) One representative case (NO.52277) with NPC provides the histological evidence for cancer pathogenesis and metastasis (Pan-cytokeratin staining). Towards central areas of primary tumors, cancer cells exhibited epithelial-like phenotypes and polarized (1). In contrast, cancer cells acquired mesenchymal-like phenotypes and lost cell polarity during tumor progression, including budding cells moving from the invasive front (2), invading the adjacent stroma (3) and the microvasculature (4). **Spindle cells might be valuable morphological predictors of “mobile/migratory CSCs” and “invasive/metastatic NPC”.**

In the last decades, numerous molecules related to tumor progression of NPC have been shown [13,48,52]. However, in spite of significant advances, the invasive and metastatic process of this disease remains less well understood. To the best of our knowledge, the integrated model of tumor cell dissemination in NPC has not yet been advanced. Of importance, using the same cohort of cancer tissues, we observed that spindle-shaped cells in the invasive front (tumor-stroma boundary) correlated strongly with EMT in NPC [16]. Consequently, we also found that this kind of cancer cells acquired stem-like features, which should be maintained by vascular niches [53,54]. Additionally, we showed that budding cells broke away from the invasive front of tumors with the potentials to migrate and invade, and commonly possessed CSCs properties [21]. On the basis of these findings, together with our current observations, a molecule-morphology model of NPC was firstly proposed to clarify the invasion-metastasis cascade of this disease. In this model (Figure 5), spindle cells with CSCs characteristics become aggressive and invasive through EMT (mainly due to EBV infection and inflammatory factors [49-51,55,56]) to dissociate from the primary sites (tumor budding), subsequently infiltrate into the surrounding matrix, and ultimately spread to lymph nodes or other distant organs to seed new tumors after entering the circulation. Different from other head and neck cancers, NPC patients have a predilection for regional lymph nodal metastasis when diagnosed and have tumor recurrence and distant metastasis after treatment. The model of spindle cells, which generates CSCs and EMT properties, is likely to account for the predominant clinical characteristics of this disease. In a whole, we demonstrate that neoplastic spindle cells might be valuable morphological predictors of tumor cell dissemination and metastasis. The identification of more specific markers of spindle cells and further understanding of the functional role of these cells should help to provide effective therapies for patients with NPC. 

## Supporting Information

Table S1 **Overview of the primary antibodies used for study.**
(DOC)

Table S2 **High expression of CSCs and EMT-related markers and high grade of spindle cells in two main cluster subgroups.**
(DOC)

Table S3 **The results for ALDH1, SOX2, OCT4, Nanog, LMP1 and EBER in 115 neoplastic spindle cells and 47 non-spindle cells of NPC.**
(DOC)
