# Correction: Molecular Characterization and Clinical Implications of Spindle Cells in Nasopharyngeal Carcinoma: A Novel Molecule-Morphology Model of Tumor Progression Proposed

**DOI:** 10.1371/annotation/8c396c9d-2e95-454e-8e4d-1434556135a0

**Published:** 2014-01-23

**Authors:** Weiren Luo, Kaitai Yao

There is an error in the in-text citation listed in the seventh sentence of the last paragraph in the discussion section. The full sentence with a corrected citation is: "Additionally, we showed that budding cells broke away from the invasive front of tumors with the potentials to migrate and invade, and commonly possessed CSCs properties [26]."

