# Supplementary material for: Molecular Characterization and Clinical Implications of Spindle Cells in Nasopharyngeal Carcinoma: A Novel Molecule-Morphology Model of Tumor Progression Proposed
Source: PLoS One. 2013 Dec 12;8(12):e83135. doi: 10.1371/journal.pone.0083135 (PMC3861507; doi:10.1371/journal.pone.0083135)
Supplement: Table S1 — Overview of the primary antibodies used for study. (DOC) [file pone.0083135.s001.doc]

| **Table S1.** Overview of the primary antibodies used for study | | | | | | |
| --- | --- | --- | --- | --- | --- | --- |
| Antigen | Source | Clone | Dilution | [Localization](http://www.google.com.hk/search?hl=en&safe=active&biw=1349&bih=608&sa=X&ei=PQTeTZ2dIIjYuAPDuMzJBQ&ved=0CBYQBSgA&q=localization&spell=1) | Positive controls | Negative controls |
| Pan-cytokeratin | Zymed | AE1/AE3 | 1:250 | Cytoplasm | Lung cancer | PBS |
| E-cadherin | BD Biosciences | 36/E-cadherin | 1:300 | Membrane | Lung cancer | PBS |
| β-catenin | Cell signaling | Ser37 | 1:150 | Cytoplasm | Lung cancer | PBS |
| N-cadherin | Zymed | clone 3B9 | 1:50 | Cytoplasm/Nucleus | Lung cancer | PBS |
| Vimentin | BD Biosciences | RV202 | 1:200 | Nucleus | Lung cancer | PBS |
| Fibronectin | Abcam | IST-9 | 1:300 | Cytoplasm | Breast cancer | PBS |
| MMP-2 | Abcam | CA-4001/CA719E3C | 1:20 | Cytoplasm | Lung cancer | PBS |
| Periostin | Abcam | - | 1:500 | Cytoplasm/Nucleus | Breast cancer | PBS |
| SPARC | Abcam | MM0557-8N38 | 1:500 | Cytoplasm/Nucleus | Breast cancer | PBS |
| Snail | Cell signaling | C15D3 | 1:100 | Cytoplasm/Nucleus | Lung cancer | PBS |
| Slug | Cell signaling | C19G7 | 1:50 | Cytoplasm/Nucleus | Lung cancer | PBS |
| ALDH1 | Abcam | EP1933Y | 1:250 | Cytoplasm | Lung cancer | PBS |
| SOX2 | Santa Cruz | E-4 | 1:300 | Nucleus | Lung cancer | PBS |
| OCT4 | Zymed | C-10 | 1:50 | Nucleus | Lung cancer | PBS |
| Nanog | Cell Signaling | 1E6C4 | 1:500 | Cytoplasm/Nucleus | Lung cancer | PBS |
| ABCG2 | Santa Cruz | BXP-21 | 1:100 | Membrane | Breast cancer | PBS |
| BMI-1 | Upstate Biotechnology | F6 | 1:100 | Cytoplasm/Nucleus | Lung cancer | PBS |
| Survivin | Cell signaling | 71G4B7 | 1:400 | Nucleus | Colorectal cancer | PBS |
| LMP1 | Santa Cruz | CS1/2/3/4 | 1:25 | Cytoplasm/Nucleus | Hodgkin lymphomas | PBS |
