# Supplementary material for: Molecular Characterization and Clinical Implications of Spindle Cells in Nasopharyngeal Carcinoma: A Novel Molecule-Morphology Model of Tumor Progression Proposed
Source: PLoS One. 2013 Dec 12;8(12):e83135. doi: 10.1371/journal.pone.0083135 (PMC3861507; doi:10.1371/journal.pone.0083135)
Supplement: Table S2 — High expression of CSCs and EMT-related markers and high grade of spindle cells in two main cluster subgroups. (DOC) [file pone.0083135.s002.doc]

| **Table S2.** High expression of CSCs and EMT-related markers and high grade of spindle cells in two main cluster subgroups | | | | |
| --- | --- | --- | --- | --- |
| Variables* |  | Cluster subgroups | | |
|  |  | Cluster A (*n* = 68) | Cluster B (*n* = 54) | χ^2^ test |
| ALDH1 |  | 15 (22%) | 33 (61%) | *P* < 0.001 |
| SOX2 |  | 23 (34%) | 45 (83%) | *P* < 0.001 |
| OCT4 |  | 16 (24%) | 27 (50%) | *P* = 0.002 |
| Nanog |  | 29 (43%) | 42 (78%) | *P* < 0.001 |
| ABCG2 |  | 19 (28%) | 39 (72%) | *P* < 0.001 |
| E-cadherin (Membrane) |  | 22 (31%) | 5 (10%) | *P* = 0.005 |
| E-cadherin (Cytoplasm) |  | 18 (27%) | 28 (52%) | *P* = 0.004 |
| N-cadherin |  | 13 (19%) | 42 (79%) | *P* < 0.001 |
| β-catenin (Cytoplasm) |  | 14 (21%) | 26 (48%) | *P* = 0.001 |
| Fibronectin |  | 21 (31%) | 28 (52%) | *P* = 0.019 |
| MMP-2 |  | 24 (35%) | 33 (61%) | *P* = 0.005 |
| Periostin |  | 28 (41%) | 42 (78%) | *P* < 0.001 |
| SPARC |  | 29 (43%) | 43 (80%) | *P* < 0.001 |
| Snail |  | 15 (22%) | 45 (83%) | *P* < 0.001 |
| Slug |  | 17 (25%) | 36 (67%) | *P* < 0.001 |
| Spindle cells (≥20%) |  | 17 (25%) | 35 (65%) | *P* < 0.001 |
| Abbreviations: CSCs, cancer stem cells; EMT, epithelial-mesenchymal transition; ALDH1, aldehyde dehydrogenase 1.  * High expression for CSCs and EMT-related markers defined as a score in all tumor cells of >6;  Spindle cells ≥ 20% were regarded as tumors with high grade of spindle cells. | | | | |
