# Supplementary material for: Molecular Characterization and Clinical Implications of Spindle Cells in Nasopharyngeal Carcinoma: A Novel Molecule-Morphology Model of Tumor Progression Proposed
Source: PLoS One. 2013 Dec 12;8(12):e83135. doi: 10.1371/journal.pone.0083135 (PMC3861507; doi:10.1371/journal.pone.0083135)
Supplement: Table S3 — The results for ALDH1, SOX2, OCT4, Nanog, LMP1 and EBER in 115 neoplastic spindle cells and 47 non-spindle cells of NPC. (DOC) [file pone.0083135.s003.doc]

| **Table S3**. The results for ALDH1, SOX2, OCT4, Nanog, LMP1 and EBER in 115 neoplastic spindle cells and 47 non-spindle cells of NPC | | | | | | |
| --- | --- | --- | --- | --- | --- | --- |
| Antigen | *P* value* | Neoplastic spindle cells  (*n*, %) | |  | Non-spindle cells  (*n*, %) | |
|  |  | Low | High |  | Low | High |
| ALDH1 | 0.000 | 24 (21) | 91 (79 ) |  | 34 (72) | 13 (28) |
| SOX2 | 0.000 | 44 (38) | 71 (62) |  | 37 (79) | 10 (21) |
| OCT4 | 0.000 | 66 (57) | 49 (43) |  | 40 (85) | 7 (15) |
| Nanog | 0.000 | 36 (31) | 79 (69) |  | 32 (68) | 15 (32) |
| Survivin | 0.000 | 19 (17) | 96 (83) |  | 37 (79) | 10 (21) |
| LMP1 | 0.000 | 58 (50) | 57 (50) |  | 38 (81) | 9 (19) |
| EBER | 0.000 | 41 (37) | 74 (63) |  | 32 (68) | 15 (32) |

*** Neoplastic spindle cells vs. non-spindle cells,*P* < 0.05 statistically significant.
